# Bibliometric analysis of the African Health Sciences' research indexed in Web of Science and Scopus

**DOI:** 10.4314/ahs.v22i2.80

**Published:** 2022-06

**Authors:** Taha Hussein Musa, Joseph Kawuki, Hassan Hussein Musa

**Affiliations:** 1 Key Laboratory of Environmental Medicine Engineering, Ministry of Education, Department of Epidemiology and Health Statistics, School of Public Health, Southeast University, Nanjing, 210009, Jiangsu Province, China; 2 Biomedical Research Institute, Darfur University College, Nyala, South Darfur, Sudan; 3 Centre for Health Behaviours Research, Jockey Club School of Public Health and Primary Care, Faculty of Medicine, The Chinese University of Hong Kong, Hong Kong - SAR, China; 4 Key Laboratory of Environmental Medicine Engineering, Ministry of Education, Global Health School of Public Health, Southeast University, Nanjing, 210009, Jiangsu Province, China; 5 Faculty of Medical Laboratory Sciences, University of Khartoum, Sudan

**Keywords:** Bibliometric analysis, African Health Sciences, VOSviewer, Publication progress

## Abstract

**Background:**

The Journal of African Health Sciences (AHS) is an internationally refereed journal in the field of health sciences with vast research contributions in the world and Africa region.

**Objective:**

The study aimed to document the scientific production and explore the AHS research bibliometric characteristics since its first issue.

**Methods:**

A comprehensive retrospective bibliometric analysis was performed on AHS published documents indexed in Web of Science (WoS) and Scopus since the journal's first issue. The analysis was done using SPSS v. 22.0, Bibliometrix Package in R, and VOSviewer v.1.6.15.

**Results:**

A total of 1649 and 1879 documents indexed in Web of Science (WoS) and Scopus were retrieved. The annual number of publications showed a significant increase in both databases. The most contributing countries (in WoS vs. Scopus) were; Nigeria (n = 393 vs. 276), Uganda (215 vs. 220) and South Africa (143 vs. 101).

The most productive authors were “Tumwine JK”, “Mayanja-Kizza H”, and “Ocama P”. Makerere University, The University of Ibadan, and University of Nigeria were the most contributive institutions. International agencies mainly from the USA were the main funders of AHS documents. Analysis of keywords revealed the dominance of research topics with keywords such as HIV/AIDS, Tuberculosis, Malaria, Obesity, Hypertension, risk factors, infection, mortality, amongst others.

**Conclusions:**

This analysis has revealed the progress in the development and growth of scientific research from AHS. Moreover, top-cited documents-analysis has reflected its focus on health issues relevant to Africa. This analysis would help in evidence-based descriptions of AHS research output.

## Introduction

African Health Sciences (AHS) with Electronic International Standard Serial Number (eISSN) 1680-6905 is an internationally refereed journal and publishes four issues per year in areas related to medicine with a focus on different research fields, including clinical practice, public health, policy, planning, implementation and evaluation. In addition, the AHS publishes health issues with science topics relevant to the Africa region. It further advocates for and promotes the growth and developments of scientific research in the Africa region throughout promoting the culture in sub-Saharan Africa, and provides high-quality services for researchers in Africa and worldwide. Throughout its history, AHS acknowledges the support provided by the African Health Journals Partnership Program that is funded by the US National Library of Medicine at the US National Institutes of Health (https://africanhealthsciences.org/authors/) with additional support from the US Fogarty International Center, the Council of Science Editors, and others. This composes the African Journal Partnership Program (AJPP) that aims to strengthen the ability of African journal editors to disseminate scientific findings more broadly to the world.

The AHS published the first 2 issues in the year 2001, followed by 3 issues per year in 2002, 2003 and 2004. Since the year 2005 AHS has published 4 times a year by the Faculty of Medicine, Makerere University, Uganda. AHS is indexed in many prestigious databases (Medline/Pubmed; Pubmed Central; African Index Medicus; Hinari; Bioline; AJOL; Science Citation Index - Clarivate).

In 2001 AHS got indexed in Scopus databases, and in 2008 it was indexed in the Web of Science Core Collection, which is the world's original citation index for scientific and scholarly research with leading citation databases covering over 12,000 of the highest impact journals worldwide and 21,100+ unique global journals (Web of Science Core Collection - Web of Science platform - LibGuides at Clarivate Analytics).

In recent years, bibliometric citation analysis has been used to identify the most influential articles published. Therefore, its use in the quality assessment of the published articles in scientific journals is necessary^1^. Indexation of a journal in many scientific databases also reflects its quality compared to other journals^2^.

Currently, there have been very few literature regarding the bibliometric analysis of scientific journals^3^,^4^,^5^, with none done for AHS. Therefore, the study aimed at identifying the scientific production and to explore the AHS journal's bibliometric characteristics during its history from the following perspectives; the hottest topics, most impactful papers, temporal distribution, most influential institutions, keywords distribution, countries of origin, authors as well as their collaboration network. The analysis would help explore various bibliometric questions and assess the contribution of various authors, countries and institutions through co-authorship analysis. We believe that conducting a bibliometric analysis of the articles published in AHS venues can not only provide insight into current publication practice but also inform about the temporal evolution of the publication trends, hot research topics and keywords frequency used by the researchers. Besides, it would provide the readership with a scientific knowledge map of the published documents in AHS during the journal's istory.

## Methods

### Search strategy and keywords

Bibliometric analyses of AHS were performed using the Web of Science Core Collection (WoS) (https://webofknowledge.com/) and Scopus (https://www.scopus.com/) online databases.

We downloaded retrieved documents from Scopus and WoS, including documents indexed in Science Citations index Expand (SCI-E) and Social Science Citations Index (SSCI) of Web of Science (Access date: 24th November 2020). The following search strategy was used in WoS: Publication name as “source titles”= (African Health Sciences) or eISSN= (1680-6905), Indexes = (SCI-Expanded, SSCI) include both (SSCI and SCI-E), Timespan = (2008–2020). The following search strategy was used in the Scopus database to search relevant documents: A unique identification number assigned to all serial publications in AHS as “ISSN (1680-6905)”, Timespan= (2001–2020).

### Inclusion criteria

The study included all documents, regardless of their type (articles, proceedings, corrections, editorials, letters or research notes, reviews and short surveys), published in WoS and Scopus. Both databases are suitable and unique, providing the most influential and reliable information that can be used in bibliometric analyses. Besides, the main information regarding the collected metadata on AHS is presented in Table 1.

**Table 1:** Main information regarding the metadata on *African Health Sciences*

Description	(Web of Science)	(Scopus)
Time span	2008:2020	2001:2020
Documents	1649	1879
Average years from publication	5.21	5.9
Average citations per document	6.409	7.968
Average citations per year per doc	0.9769	1.065
References	41091	49325
Document types, n (%)		
Article	1508 (91.45)	1730 (92.07)
Proceedings paper	7 (0.42)	37 (1.97)
Correction	2 (0.12)	3 (0.16)
Editorial	59 (3.58)	31 (1.65)
Letter or research note	37 (2.24)	20 (1.06)
Review	36 (2.18)	56 (2.98)
Short survey	0 (0.0)	2 (0.11)
Document contents		
Keywords Plus	3135	11829
Author's Keywords	4242	4378
Authors		
Authors	5890	6504
Author Appearances	7105	8009
Authors of single-authored documents	110	149
Authors of multi-authored documents	5780	6355
Authors Collaboration		
Single-authored documents	159	205
Documents per Author	0.28	0.289
Authors per Document	3.57	3.46
Co-Authors per Documents	4.31	4.26
Collaboration Index (CI)	3.88	3.80

### Data Extraction

The relevant data were extracted from the WoS (https://webofknowledge.com/), and Scopus (https://www.scopus.com/) webpages, which was provided by the Southeast University library (https://www.seu.edu.cn/). Data search and extraction were performed independently by two researchers (THM and JK). The extracted information included; the number of published documents in each database, most productive institutions, authors and countries, citation frequency scores, funding agencies and subject categories. In addition, the socioeconomic factor (Gross Domestic Product (GDP)), a determinant for the number of published articles from each country, was obtained from World Bank Open Data (https://data.worldbank.org/). The correlation analysis between GDP and annual publication number was investigated for documents published in AHS.

### Data analysis

To achieve our main objective of exploring AHS journal's bibliometric characteristics, data were analysed using Bibliometrix Package in R program, which identified the annual trends of publications, top productive authors, citation frequency, H-Index of institutions and countries, country contributions, and hot topics from top frequent keywords^6^. In addition, VOSviewer (v.1.6.15) was used to show the network visualization, bibliographic coupling, co-citation, or co-authorship relations^7^. In co-citation analysis, we used a set of highly cited references, authors, and documents in the AHS journal. We also applied text processing using terms such as title, author keywords, and keywords plus. Pearson's correlation coefficient (r) was also used to study correlations between specific variables. All statistical analyses were performed using SPSS v. 22.0 (IBM SPSS Software Inc., Chicago, Illinois, USA). P-value <0.05 was considered to be statistically significant.

## Results

### Main characteristics of the metadata

A comprehensive analysis of WoS and Scopus databases gave 1649 and 1879 documents respectively, with 6.409 average citations per document in WoS and 7.968 average citations per document in Scopus. Summaries of the document type, content, authors and authors' collaboration are shown in Table 1.

### Annual Publication trends

The annual trend of publication showed that 68 documents were published from WoS in 2008, after which the annual output gradually increased over the next years, reaching a maximum of 221 publications in 2019 (Figure 1(a)). The production trends from Scopus also followed a similar trend starting with 21 documents in 2001, with a gradual increase in recent years (Figure 1(b)). The analysis shows significant positive correlations between the number of published documents and the number of citations reported in WoS (r=0.480 (low), P=0.0114), and Scopus (r=0.547 (moderate), P=0.015). The number of total citations (NTC) ranged from 410 to 1417 with a total of 10570, and a median of 923.0 (IQR=512) for documents indexed in WoS. That from Scopus ranged from (64 to 1753), with a total of (10570), and a median of 649.0 (IQR=944) TNC.

**Figure 1.a:**
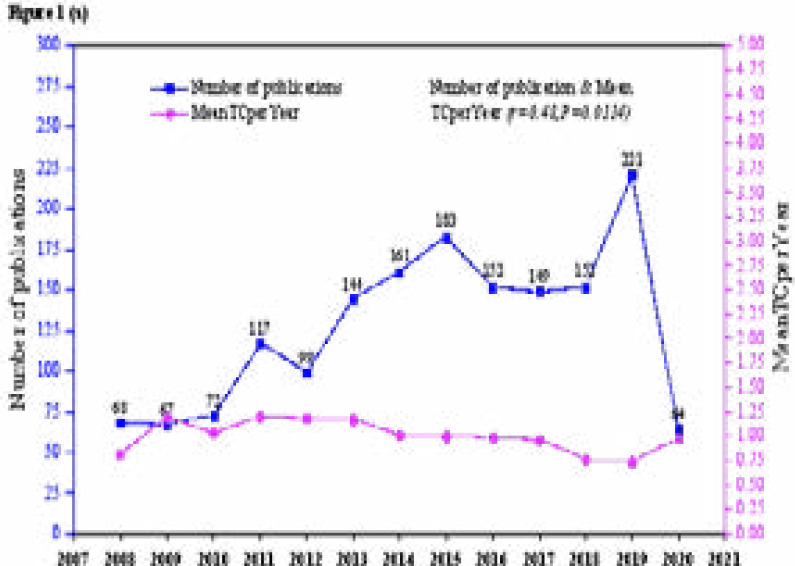
Annual growth of the number of publications and Mean of Total Citations Per Year (Mean TCperYear) for AHS documents indexed in WoS (2008 to 24^th^ November 2020).

**Figure 1.b:**
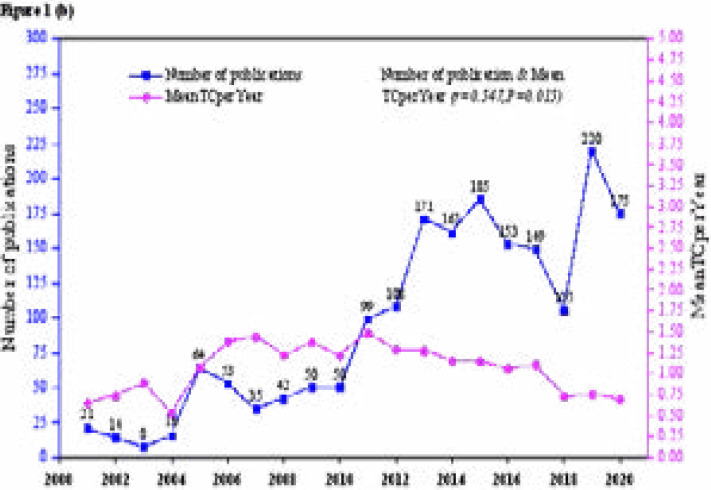
Annual growth of the number of publications and Mean Total Citations Per Year (Mean TCperYear) for AHS documents indexed in the Scopus database (2001 to 24^th^ November 2020).

### Most cited documents

Table 2 shows the top 10 documents according to the number of citations for AHS as indexed in WoS8–17 and Scopus181982021129132210. The first top cited paper in WoS is published by Cherian A and Thomas SV in 2011, with 74 citations 8. The article highlighted several important aspects regarding central nervous system tuberculosis, including its devastating clinical manifestations 8.

**Table 2:** Top 10 highly cited papers that were published in AHS between (2005–2012)

R^a^	Authors, document title, year	TNC^b^	DT^c^
	**Web of Science (WoS)**		
1	Cherian A, Thomas SV. Central nervous system tuberculosis. 2011;11(1): 116–127.	74	Article
2	Regassa N. Antenatal and postnatal care service utilization in southern Ethiopia: a population-based study.2011;11(3): 390–397.	66	Article
3	Tumwine J K. Clinical and epidemiologic characteristics of nodding syndrome in Mundri County, southern Sudan.2012; 12(3): 242–248.	64	Article
4	Beyeza-Kashesya Jolly, et al. The dilemma of safe sex and having children: challenges facing HIV sero-discordant couples in Uganda.2009;8 (1):2–12.	64	Article
5	Kang'ethe EK, Lang'a, KA. Aflatoxin B1 and M1 contamination of animal feeds and milk from urban centers in Kenya.2009; 9(4): 218–226.	62	Article
6	Mchunu Gugu, et al. Adolescent pregnancy and associated factors in South African youth. 2012. 2(4): 426–434.	61	Article
7	Urasa M, Darj E.Knowledge of cervical cancer and screening practices of nurses at a regional hospital in Tanzania.2011;11(1):48–57.	57	Article
8	Bilici S. et al. Mean Platelet volume in diagnosis of acute appendicitis in children.2011;11(3),427–432.	55	Article
9	Heydarnejad MS. Factors affecting quality of life in cancer patients undergoing chemotherapy. 2011;11(2),266–270.	55	Article
10	Nwagha, UI. Atherogenic index of plasma as useful predictor of cardiovascular risk among postmenopausal women in Enugu, Nigeria.2010;10(3): 248–252.	51	Article
	**Scopus database**		
1	Olaitan PB. et al. Honey: A reservoir for microorganisms and an inhibitory agent for microbes.2007;7(3):159–165.	124	Article
2	Orem J., et al. Burkitt's lymphoma in Africa, a review of the epidemiology and etiology.2007;7(3):166–175.	112	Article
3	Cherian A & Thomas SV. Central nervous system tuberculosis. 2011;11(1):116–127.	103	Article
4	Langen TT.Gender power imbalance on women's capacity to negotiate self-protection against HIV/AIDS in Botswana and South Africa. 2005; 5(3):188–197.	99	Article
5	Nakku JN. Et al. Postpartum major depression at six weeks in primary health care: Prevalence and associated factors.2006;6 (4):207–214.	81	Article
6	Kang'Ethe, EK, Lang'A KA.Aflatoxin B1 and M1 contamination of animal feeds and milk from urban centers in Kenya. 2009; 9 (4):218–226.	79	Article
7	Regassa N.Antenatal and postnatal care service utilization in Southern Ethiopia: A population-based study.2011;11(3):390–397.	77	Article
8	Mchunu G. Adolescent pregnancy and associated factors in South African youth.2012;12(4):426–434.	73	Article
9	Kamatenesi-Mugisha M, Oryem-Origa H. Traditional herbal remedies used in the management of sexual impotence and erectile dysfunction in western Uganda.2005;5(1):40–49.	71	Article
10	Tumwine JK.Clinical and epidemiologic characteristics of nodding syndrome in Mundri county, southern Sudan.2012;12(3):242–248.	68	Article

a Ranking

b Total number of citations

c Documents Type

In contrast, the first top cited article in Scopus was written by Olaitan PB. et al. 2007, with 124 citations^18^. The article covers the vital aspects regarding the increasing use of honey in medical and wound care^18^.

The 10 top-cited papers in WoS had a citation range of (51 to 74), with TNC (609), and a median of 61.50 (IQR=9), while those from Scopus their citation ranged from (68 to 124), with TNC (887), and a median of 80.70 (IQR=30).

### Top 10 active authors in AHs

In this study, 5890 and 6504 authors respectively contributed to the publications indexed in the WoS and Scopus databases. The 10 most prolific authors that published documents in AHS are listed in (Table 3). The most productive and cited author for AHS in WoS was Tumwine JK, who published 46 documents (including 8 articles and 38 editorials) between (2008–2020), and in Scopus with 58 documents (15 articles, 36 editorials, and 7 research notes) between (2004–2020). He was followed by Abd El-Kader SM with (n=17) documents in WoS between (2013–2019), and Ocama P with (n=17) documents indexed in the Scopus database during the years (2008–2020).

**Table 3:** Top 10 productive authors in AHS

			Web of Science					Scopus		

R^a^	Author name	h_index	(TNC)^b^	(NP)^c^	Production over time	Author name	h_index	(TNC)^b^	(NP)^c^	Production of author over time
**1**	Tumwine, J.K.	6	164	46	2008∼2020	Tumwine, J.K.	5	151	58	2004∼2020
**2**	Abd El-kader S.M.	7	110	17	2013∼2019	Ocama, P.	6	108	17	2008∼2020
**3**	Ocama, P.	6	96	17	2008∼2020	Abd El-Kader, S.M.	7	139	16	2013∼2020
**4**	AL-Jiffri, O.H.	6	97	15	2014∼2019	Abu-Zidan, F.M.	7	161	16	2006∼2018
**5**	Abu-Zidan, F.M.	5	85	13	2008∼2018	Al-Jiffri, O.H.	6	106	15	2014∼2020
**6**	Mung'ayi V	3	43	13	2013∼2019	Mayanja-Kizza H	9	172	14	2007∼2018
**7**	Mayanja-Kizza, H.	8	119	12	2008∼2018	Moselhy, S.S.	3	30	11	2015∼2020
**8**	Al-Shreef, F.M.	5	59	9	2014∼2019	Galukande, M.	7	137	10	2005∼2019
**9**	Eze, C.U.	3	32	8	2013∼2017	Mungâyi, V.	2	18	10	2015∼2019
**10**	Galukande, M.	5	81	9	2008∼2019	Okonko, I.O.	5	57	10	2012∼2020

a R, Ranking

b Total number of citations

c Number of productions documents

### Author collaboration

**Figure 2:**
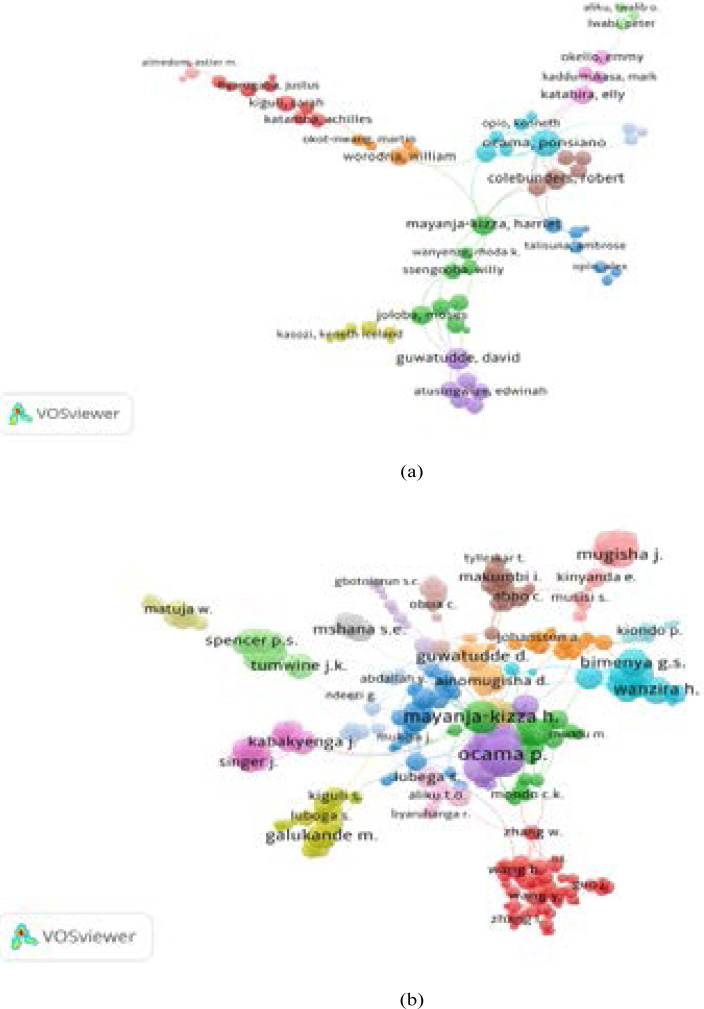
Co-authorship network analysis of the most influential authors of AHS documents indexed in WoS (a) and Scopus (b)

The collaboration index (CI) of authors for documents from WoS was 3.88 and 3.80 from Scopus. For the top productive authors, the number of links (L) and total link strength (TLS) of the co-authorship was calculated following the minimum number of publications being set as 2 authors per document. The findings show that in WoS, the author communities were presented in 12 clusters shown with different colours, all together with (L=122, TLS=162) Figure 2 (a). While in Scopus, the author collaboration was presented in 19 clusters, and with (L=780, TLS=1144) Figure 2 (b). Examples of strong collaboration in WoS were seen among the following authors: Ocama, P.(L=13, TLS=22), Guwatudde, D. (L=9, TLS=11), Mayanja-Kizza H. (l=8, TLS=8), Kamya M.R (L=5, TLS=5), and Tumwine, J.K (L=3, TLS=3). In Scopus, strong collaborations were seen among; Tumwine, J.K. (L=12, TLS= 18), Ocama, P. (L=24, TLS=51), and Bimenya, G.S. (L=13, TLS=24), and Mayanja-Kizza H. (L=12, TLS=22).

In Figure 2(a and b), the strength of collaboration is relative to the thickness of the connection between any two scientists. Authors with similar circle colours are considered as one group of clusters, i.e. have a close collaboration.

### Most productive institutions

**Figure 3:**
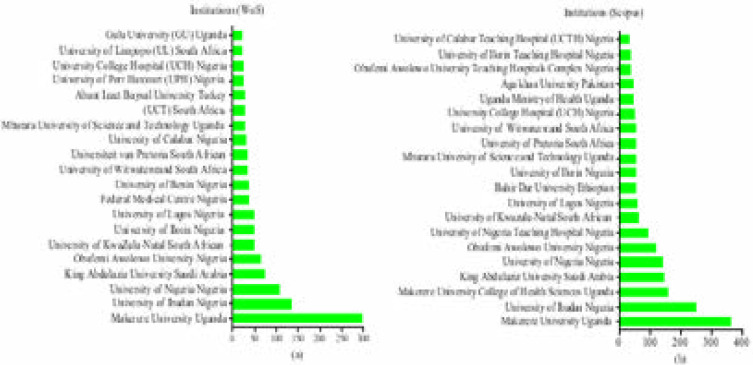
Top 20 productive institutions/organizations of AHS publications in WoS (a), and Scopus (b).

The frequency distribution of the top 20 most productive institutions in AHS are presented in Figure 3 (a and b). From both databases, these included: Makerere University-Uganda, University of Ibadan-Nigeria, University of Nigeria, King Abdulaziz University, and Obafemi Awolowo University-Nigeria, among others. Meanwhile, in Uganda, Makerere University is one of the pioneering institutions and has highly contributed to documents in AHS, starting from 2008 up to date.

### Most dominant funding sources for AHS publications

Financial support from public foundations or commercial agencies has greatly contributed to medical and public health research. Our study identified and acknowledged more than 1498and 4451 funding agencies for documents indexed in WoS and Scopus, respectively. Out of these, only the top 10 funding agencies from WoS and Scopus documents are shown in Table 4. The analysis shows the United States Department of Health Human Services funded 48 articles, and National Institutes of Health (NIH)-USA funded 46 articles indexed in WoS. Among documents indexed in Scopus, most of the funding was from National Institutes of Health (NIH)-USA, which funded 21 articles followed by Fogarty International Center funding 20 articles, among others (Table 4). The evidence level in the top 10 funding agencies revealed a significant correlation between the number of funded articles and TNC for both databases (r=1.00, p=0.0001).

**Table 4:** Top 10 funding agencies listed in AHS documents from WoS and Scopus

Funding Agencies (WoS)	NP^a^	TNC^b^	Funding Agencies (Scopus)	NP^a^	TNC^b^
United States Department of Health Human Services	48	308	National Institutes of Health (NIH), USA	21	167
National Institutes of Health (NIH), USA	46	295	NIH Fogarty International Center (FIC)	20	159
NIH Fogarty International Center (FIC)	29	186	King Abdulaziz University	13	104
Deanship of Scientific Research, King Faisal University	21	135	National Natural Science Foundation of China	9	72
Deanship of Scientific Research (DSR), King Abdulaziz University Jeddah	18	115	University of Pretoria	8	64
National Natural Science Foundation of China (NSFC)	18	115	Medical Research Council	7	56
National Research Foundation South Africa	9	58	National Institute of Neurological Disorders and Stroke	7	56
World Health Organization (WHO)	8	51	Deanship of Scientific Research, King Faisal University	6	48
Bill Melinda Gates Foundation	7	45	National Research Foundation	5	40
Deanship of Scientific Research (DSR) at King Abdulaziz University Jeddah	7	45	Deanship of Scientific Research, King Saud University	4	32

a Number of papers

b Total number of citations

### Top leading countries in published documents

The top 10 most productive countries (according to the corresponding author's country) and their citation frequencies are shown in Table 5. Papers from Nigeria had the highest citation, followed by Uganda, and South Africa. In both databases, there were significant correlations between number of articles and Single Country Publications (SCP) (r=0984, P<0.0001; r=0.867, P=0.001) and number of articles and Multiple Country Publications (MCP) (r=0.655, p=0.040; r=0.647, P=0.43) for articles indexed in WoS and Scopus database, respectively.

**Table 5:** Top 10 productive countries for AHS publications indexed in WoS and Scopus

SCR^a^	Country	Articles	(TNC)^b^	(GDP per capita) c	(SCP)^d^	(MCP)^g^	NP per GDP	SCP per GDP	MCP per GDP
	**Web of Science**								
1	Nigeria	393	2519	448.12^e^	350	43	0.877	0.781	0.096
2	Uganda	215	1378	34.387 e	141	74	6.252	4.100	2.152
3	South Africa	143	916	351.432 e	108	35	0.407	0.307	0.100
4	China	100	641	14.343^f^	92	8	6.972	6.414	0.558
5	Turkey	92	590	754.412 e	89	3	0.122	0.118	0.004
6	Saudi Arabia	57	365	792.967 e	34	23	0.072	0.043	0.029
7	USA	51	327	21.374^f^	21	30	2.386	0.983	1.404
8	Ethiopia	50	320	96.108 e	46	4	0.520	0.479	0.042
9	India	43	276	2.875^f^	41	2	14.957	14.261	0.696
10	Kenya	40	256	95.503^e^	36	4	0.419	0.377	0.042

	**Scopus database**								
1	Nigeria	275	2191	448.12^e^	252	23	0.6137	0.562	0.051
2	Uganda	202	1610	34.387 e	130	72	5.8743	3.780	2.094
3	South Africa	101	805	351.432 e	78	23	0.2874	0.222	0.065
4	China	59	470	14.343^f^	58	1	4.1135	4.044	0.070
5	Turkey	45	359	754.412 e	44	1	0.0596	0.058	0.001
6	USA	40	319	21.374^f^	15	25	1.8714	0.702	1.170
7	Saudi Arabia	39	311	792.967 e	32	7	0.0492	0.040	0.009
8	Ethiopia	32	255	96.108 e	30	2	0.3330	0.312	0.021
9	Tanzania	29	231	63.177^e^	20	9	0.4590	0.317	0.142
10	Kenya	28	223	95.503^e^	23	5	0.2932	0.241	0.052

a Standard competition ranking

b Total number of citations

c GDP per capita (Year 2019)

d Single Country Publication (intra-country collaboration)

e billion

f trillion

g Multiple Country Publications (inter-country collaboration)

When the research output was standardized by Growth Domestic Product (GDP) per capita, India ranks first (14.957) documents per GDP, followed by China (6.972), Uganda (6.252) for articles indexed in WoS. Where Uganda with (5.8743) documents per GDP, followed by China (4.044) ranked first for documents published in Scopus.

### Country collaboration

**Figure 4:**
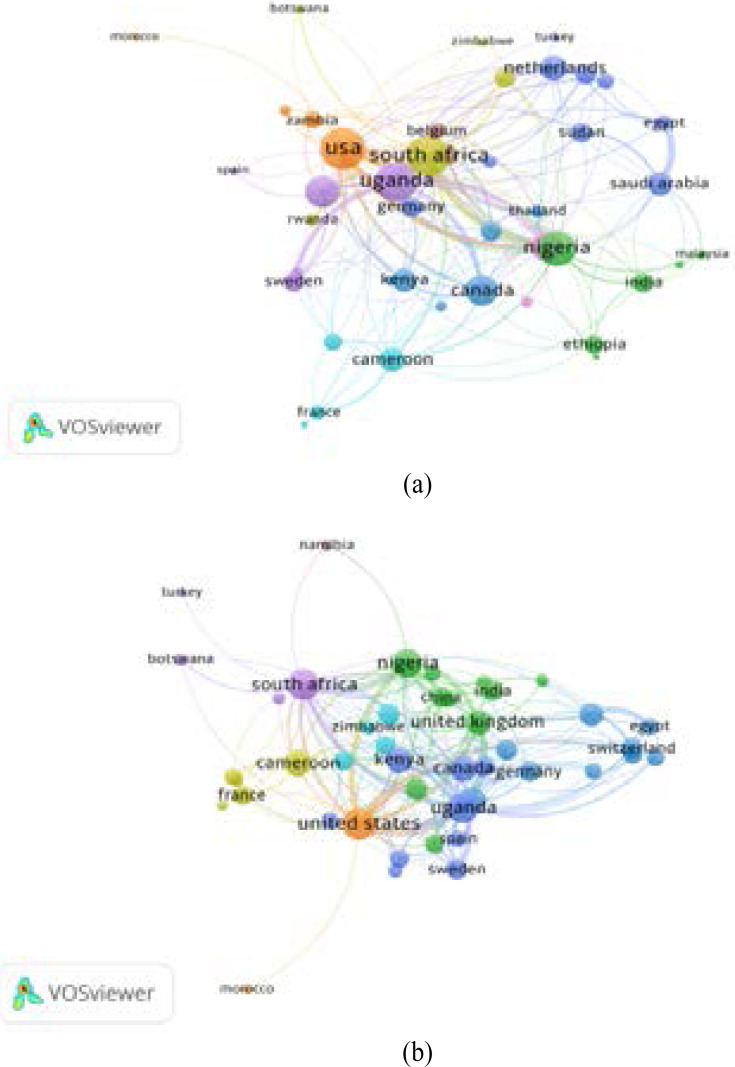
Co-authorship network analysis of the most productive countries for AHS documents from WoS (a) and Scopus (b).

The retrieved articles were from 99 countries for papers from WoS and 121 countries in the Scopus database. A minimum of 5 documents per country was set, which resulted in 43 countries meeting the threshold in WoS and 46 in Scopus. For WoS, the countries are distributed within 9 clusters with links (L)=199 and total link strength (TLS)=552. In Figures 4 (a and b), the strength of collaboration is relative to the thickness of connection between any two countries, and countries with similar circle colours are considered as one cluster, i.e. have a close collaboration. Moreover, larger nodes indicate a higher publication count; thus, smaller nodes are countries with low publication counts. The most significant collaborations in WoS documents are seen in Uganda (L=26, TLS=157), the USA (L=28, TLS=129), South Africa (L=27, TLS=102), Nigeria (L=22, TLS=98), Saudi Arabia (L=12, TLS=37), Tanzania (L=19, TLS=40), Sweden(L=10, TLS=27), Netherlands (L=12, TLS=20), India (L=10, TLS=16), and Sudan (L=10, TLS=18) among others, as shown in Figure 4 (a). For Scopus, strong collaboration was seen among the following countries: Uganda (L=26, TLS=183), the USA (L=29, TLS=150), South Africa (L=27, TLS=113), Nigeria (L=22, TLS=104), United Kingdom (L=16, TLS=81), Cameron (L=17, TLS=33), Saudi Arabia (L=13, TLS=39), and Sudan (L=8, TLS=20), as shown Figure 4 (b).

### Frequency distribution of keywords

**Figure 5:**
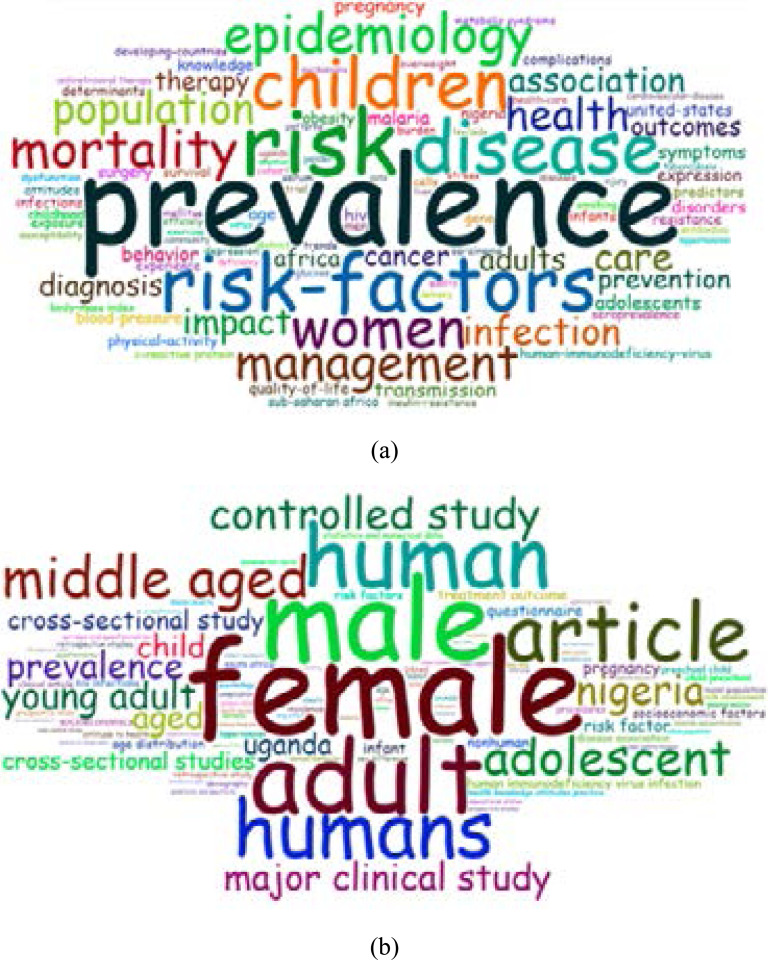
Word-cloud visualization of the 100 most-frequent Keyword Plus in AHS documents indexed in WoS (a) and Scopus (b).

According to the mean frequency of appearance for Authors' Keywords, the distribution of keywords is presented in Table 6. Topics of keywords such as; ‘Nigeria’, ‘Uganda’, ‘HIV’, ‘Prevalence’, ‘Risk factors,’ and ‘Tuberculosis’, among others had the most occurrence in AHS documents from both databases. In addition, the Word-clouds of keyword Plus for documents from both databases confirmed the knowledge structure of the common research topics published in AHS, as shown in Figure 5 (a and b). In the Word-cloud visualization, the size and centrality of the word reflect its frequency and dominance.

**Table 6:** Top 20 most frequently used Authors' keywords

Authors Keywords (WoS)				Authors Keywords (Scopus)			

Terms	Frequency	Terms	Frequency	Terms	Frequency	Terms	Frequency
Nigeria	107	Hypertension	21	Nigeria	116	Obesity	24
Uganda	71	South Africa	20	Uganda	78	Africa	22
HIV	60	Ethiopia	19	HIV	68	Ethiopia	22
Prevalence	39	Tanzania	19	Prevalence	39	Hypertension	20
Risk factors	35	HIV/AIDS	17	Children	38	Malaria	19
Children	32	Diabetes	16	Risk factors	36	Tanzania	19
Tuberculosis	25	Malaria	16	South Africa	30	Diabetes	17
Knowledge	24	Ghana	15	Tuberculosis	27	Ghana	17
Africa	22	Type 2 diabetes	15	HIV/AIDS	24	Adolescents	16
Obesity	22	Adolescents	14	Knowledge	24	Epidemiology	16

## Discussion

Our bibliometric analysis provided a comprehensive overview of the AHS journal since its first issue and this has shed light on the scientific publication patterns, including published research focus, most productive authors, institutions, and countries, basing on WoS and Scopus databases. In general, the number of AHS documents in both databases has gradually increased, implying a significant scientific research output. A similar upward trend has also been reported in bibliometric studies of other journals 4,5.

The study revealed the characteristics of the top 10 cited articles from AHS indexed in WoS and Scopus. The top-cited documents discussed various key health issues relevant to the African population, including infectious diseases, vectors, cardiovascular disease, and community health. From Scopus, the most highly cited article was a review written by Olaitan PB. et al., 2007, where the authors discussed the conventional anti-microbial properties of honey and its use in the treatment of wounds^18^. Given the interesting aspects discussed in this article, that is, the use of honey both as a food and a remedy, it makes this article more relevant to community healthIn addition, this paper was published quite a long-time ago within the first issues of AHS journal, which also explains its high citation score. From WoS, the most influential paper based on the total citation was written by Cherian A and Thomas S.V, 2011 under the title “Central nervous system tuberculosis” with 74 citations^3^. This review paper discussed central aspects of tuberculosis (TB), including its prevalence, epidemiology, its devastating complications, treatment and management, among others. Given the relevance, in terms of burden, of TB in Africa and globally, this paper no-wonder attracted many citations, making it the highest cited article of AHS 23.

Other highly cited AHS articles, addressed issues such as; antenatal and postnatal care service^9^, nodding syndrome^10^, the dilemma of safe sex and having children^11^, Aflatoxin B1 and M1 contamination^12^, cervical cancer^14^, Adolescent pregnancy^13^, Quality of life in cancer patients^16^, cardiovascular risk among postmenopausal women^17^, epidemiology and aetiology of Burkitt lymphoma in Africa region^19^, postpartum major depression and its complications^21^, among others.

The identification of top-cited articles in AHS is useful as it reflects the diverse scope and health research fields that are of high interest to the readership of AHS, in addition to helping readers know the authors and institutions that have highly contributed to such influential studies.

A previous study showed that highly cited publications are usually involving due to their international collaboration as they are often authored by several researchers^24^. Moreover, highly cited articles are very different from ‘ordinary’ cited articles^25^. Since citation is used as a key indicator of research quality, highly cited publications are positively correlated with the h-index of the author, institution and country^26^. We found that the h-index for AHS publications was 40, which is less than that of the Asian Pacific Journal of Tropical Biomedicine^4^ but was slightly higher than that of several other scientific journals^5^.

Our study also explored the changing annual trends in co-authorship over the history of AHS, which highlighted not only the most active authors but also the authors' production timeline in AHS over the study period. Previous studies show that author citation analysis is a good factor to evaluate the impact and usability of research done by that researcher 27,28. In our analysis, we noticed that based on the number of articles and citation score, authors from African countries dominated the list. This could imply the usability and relevance of the AHS journal to the African research community. Out of the high prolific authors in AHS, 9 had more than ten articles.

The analysis of the most productive countries in AHS publications revealed Nigeria, as the leading country based on single country papers, followed by Uganda and South Africa. Concerning the number of publications relative to GDP per Capita, Uganda, China, Nigeria, and India were the outstanding productive and influential countries of AHS research. This could certainly be due to their huge population and funding allocation by the respective governments to key research projects. Nevertheless, Uganda, the USA, South Africa, and Nigeria are the main players in international collaboration in the scientific research network. Notably, most of the top contributing countries are developing countries with the collaboration of some developed countries; African countries contributed 1002/1585 (63.21%) articles. This indicates that AHS is committed to publishing, promoting and enhancing African research.

The analysis showed that, although most of the research in AHS comes from African institutions, the funding of this research mainly comes from non-African agencies including the US Department of Health Human Services, NIH, among others. Moreover, there was a significant degree of collaboration noted among several African countries and institutions with other countries, especially the key funders. This implies that non-African countries not only finance but are also actively involved and interested in African research.

The analysis of keywords revealed that AHS studies covered various essential health aspects, including the prevalence, risk factors, outcomes and management of key endemic diseases, as well as vulnerable groups affected like children and women. Infection is also highlighted as the keyword with the most co-occurrence in the AHS journal, and this reflects the spectrum of health issues of public health importance within the region, as well as the scope of research topics of interest to the AHS readership.

Finally, the overall analysis of the most cited articles, keywords, most productive countries and institutions are a vital reflection of AHS journal's scope, aims and focus; as it advocates for and promotes the growth and development of scientific research in the Africa region as well as promoting health in Sub-Saharan Africa.

## Implications of study findings

Considering the findings of this study, it is evident that AHS cautiously advocates for and promotes the growth and development of research in the Africa region. The journal further provides high-quality services and continuously share with the scientific community publications relevant to health issues of concern from Africa and worldwide.

With the dominance of publications on topics like HIV/AIDS, Tuberculosis, Malaria, Obesity, and Hypertension, risk factors, among others, the journal is committed to improving the health of African communities.

## Limitations of the study

Although we used WoS and Scopus databases to assess scientific production and track the citation record of AHS published documents, other databases such as Google scholar were not included in the search because it is not possible to identify the annual number of reported citations of the published documents. Therefore, the results of our study might not be comprehensive^29^. Secondly, not all citations are the same, for instance, there are negative citations that draw attention to flaws found in a specific paper^30^. In addition, the older publications in any journal tend to have a higher number of citations regardless of their scientific impact than the recently published papers. Moreover, low to moderate correlations were observed for the number of published documents and the number of citations. Despite all these limitations, this study provides some insights into the most influential articles and AHS performance during its lifetime.

## Conclusions

This current bibliometric analysis has highlighted a significant increase in AHS scientific production since its first issue, and over time, there has been growth in the number of publications with a significant positive correlation with the citation number. Moreover, the analysis showed that the articles published in AHS cover abroad range of health topics, providing a contemporary overview of public health issues of enhanced research in Africa. Besides, the study revealed that AHS publications came mainly from African institutions and countries; but the journal also continuously published significant research from other countries worldwide. There were also noticeable collaborations among the authors, institutions, and countries publishing research in AHS. However, AHS studies are funded mainly by foreign agencies, with little funds from Africa. Nevertheless, AHS continues to be dedicated, through regular issues, to advocating for and promoting the growth and development of scientific research within Africa and globally.
